# From an Alternative Medicine to a New Treatment for Refractory Epilepsies: Can Cannabidiol Follow the Same Path to Treat Neuropsychiatric Disorders?

**DOI:** 10.3389/fpsyt.2021.638032

**Published:** 2021-02-11

**Authors:** Rafael M. Bitencourt, Reinaldo N. Takahashi, Elisaldo A. Carlini

**Affiliations:** ^1^Laboratory of Behavioral Neuroscience, Graduate Program in Health Sciences, University of Southern Santa Catarina, University of Southern Santa Catarina (UNISUL), Tubarão, Brazil; ^2^Post Graduate Program in Pharmacology, Department of Pharmacology, Federal University of Santa Catarina, Federal University of Santa Catarina (UFSC), Florianópolis, Brazil; ^3^Centro Brasileiro de Informações Sobre Drogas Psicotrópicas (CEBRID), Department of Preventive Medicine, Federal University of São Paulo, UNIFESP, São Paulo, Brazil

**Keywords:** cannabis, phytocannabinoids, cannabidiol, epilepsy, neuropsychiatric disorders

## Abstract

Although cannabis has been known for ages as an “alternative medicine” to provide relief from seizures, pain, anxiety, and inflammation, there had always been a limited scientific review to prove and establish its use in clinics. Early studies carried out by Carlini's group in Brazil suggested that cannabidiol (CBD), a non-psychotropic phytocannabinoid present in *Cannabis sativa*, has anticonvulsant properties in animal models and reduced seizure frequency in limited human trials. Over the past few years, the potential use of cannabis extract in refractory epilepsy, including childhood epilepsies such as Dravet's syndrome and Lennox-Gastaut Syndrome, has opened a new era of treating epileptic patients. Thus, a considerable number of pre-clinical and clinical studies have provided strong evidence that phytocannabinoids has anticonvulsant properties, as well as being promising in the treatment of different neuropsychiatric disorders, such as depression, anxiety, post-traumatic stress disorder (PTSD), addiction, neurodegenerative disorders and autism spectrum disorder (ASD). Given the advances of cannabinoids, especially CBD, in the treatment of epilepsy, would the same expectation regarding the treatment of other neuropsychiatric disorders be possible? The present review highlights some contributions from Brazilian researchers and other studies reported elsewhere on the history, pre-clinical and clinical data underlying the use of cannabinoids for the already widespread treatment of refractory epilepsies and the possibility of use in the treatment of some neuropsychiatric disorders.

## Introduction

*Cannabis sativa*, a plant popularly known for giving rise to marijuana, has in its composition more than 140 compounds called phytocannabinoids. In addition to the phytocannabinoids present in the plant, endocannabinoids (eCB) are produced endogenously through physiological stimulation and cannabinoids of synthetic origin, all called cannabinoids. Both together and isolated, cannabinoids have a wide variety of effects on the nervous system, making these compounds promising psychopharmacological alternatives in treating many neuropsychiatric disorders (1–3). Among the possibilities for pharmacotherapeutic use, stand-depression, anxiety, post-traumatic stress disorder (PTSD), addiction, neurodegenerative disorders, autism spectrum disorder (ASD), and especially refractory epilepsy, among others (4–12).

Concerning the treatment of refractory epilepsies, the last few years have shown a significant increase in studies evaluating the risks and benefits of using cannabinoids in this context (13, 14). Epilepsy is a pathological condition that affects about 65 million people worldwide, and its principal characteristic is recurrent seizures, and its etiology can be varied, ranging from genetic syndromes to brain damage (15–18). It is also a condition that often does not respond to the pharmacotherapy used, and, in this sense, cannabinoids appear as a promising alternative. The two phytocannabinoids most researched for the treatment of epilepsies are delta-9-tetrahydrocannabinol (THC—main psychoactive compound) and especially cannabidiol (CBD—main non-psychoactive compound), which are useful in preventing seizures and reducing mortality, with low toxicity and high tolerability (11, 17–20). The path to the safe and effective use of cannabinoids in treating epilepsy seems to be unraveled by science; however, the next question: would the same expectation regarding the treatment of other neuropsychiatric disorders be possible? To shed light on this issue, this review, in addition to emphasizing the use of CBD in the treatment of epilepsy, examines the possibility of using this compound as an alternative to the treatment of some neuropsychiatric disorders. For more details about the botany, psychobiology, and the medical potential of cannabis, the readers can examine the various reviews available in the literature or direct toward an elegant review by Solymosi and Köfalvi (21).

## “From an Alternative Medicine:” First Evidence and Carlini's Group Contribution

The use of Cannabis for the treatment of epilepsy has been going on for a long time, with evidence found in Sumerian tablets more than 3,800 years ago (14). The most recent reports started in the middle of the nineteenth century when the Irish surgeon William O'Shaughnessy announced the plant's therapeutic effects in the treatment of epilepsy, a fact that was soon reinforced by two other renowned English neurologists, J. R. Reynolds and W. Gowers (22, 23). Scientific publications from the 1940s, both in animal models (24) and in children with epilepsy (25), were the first reports of the therapeutic use of Cannabis for this condition. A significant step in the study of cannabinoids was taken by Mechoulam in the 1960s, when he isolated, clarified the structure, and synthesized THC and CBD, the most abundant and most studied phytocannabinoids in Cannabis to date, including for epilepsy (26–28).

In the sequence, and even before the discovery of the eCB system (which occurred only in the '90s), the Brazilian researcher Elisaldo Carlini started studies using CBD in animal models of epilepsy, suggesting the first scientific evidence about the therapeutic potential of CBD in treatment of this pathology (29, 30). Next, in partnership with the Mechoulam group, Carlini et al. conducted the first placebo-controlled study of CBD in patients with refractory epilepsy. At the time, the authors showed that two of the four epileptic patients treated with 200 mg of CBD daily showed an improvement in their epileptic status, without having any seizures within 3 months of treatment. The third patient had a partial improvement, while the fourth patient treated with CBD, and the other five patients in the placebo group showed no improvement. No toxic effects were observed, and this was the first study in humans from the “modern scientific era” to consider the possibility of CBD's therapeutic potential, isolated, in the treatment of refractory epilepsies (31).

Continuing the investigations, Carlini et al. published a series of studies that confirmed CBD's therapeutic potential in the treatment of seizures. In the early 1980s, a double-blind controlled trial was performed with CBD 200–300 mg/kg or placebo administered daily over more than 4 months in 15 patients with generalized epilepsy. Of the eight patients treated with CBD, four of them had practically no seizures throughout the experiment, and three had partial improvement, while the seven patients in the placebo group showed no improvement in the clinical picture of the seizures (32).

**Figure 1:**
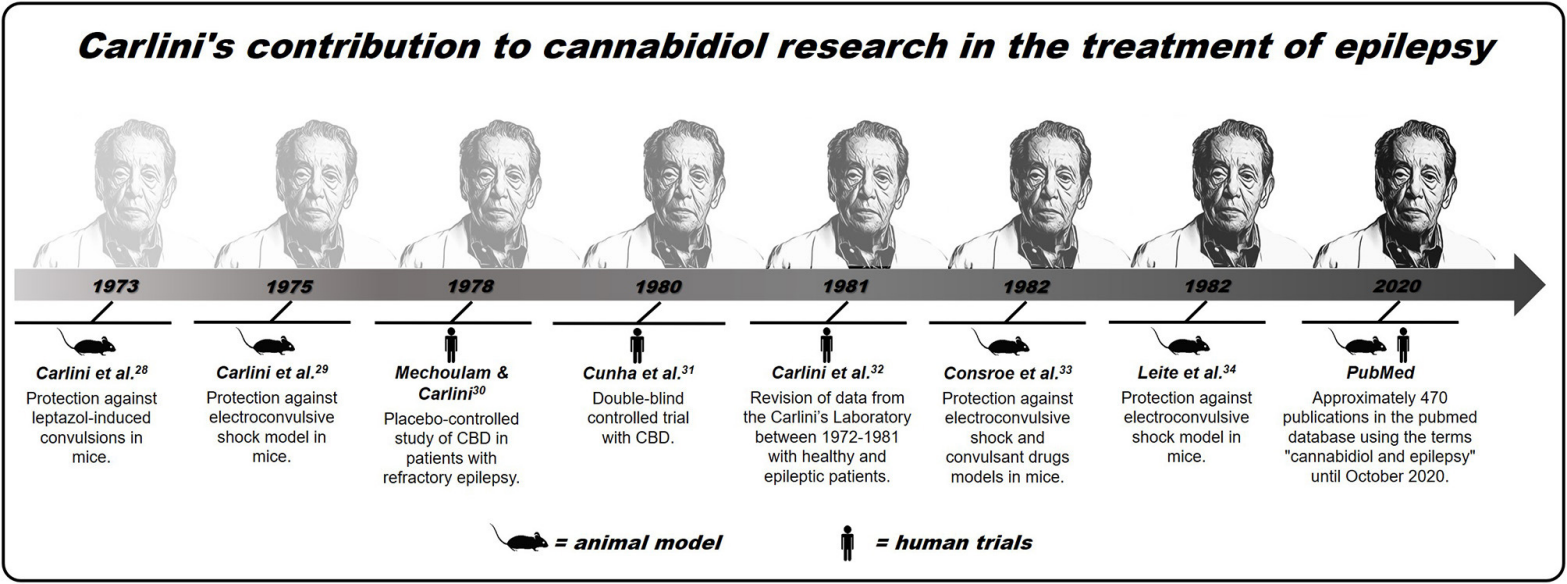
Brief history of Carlini's contribution to cannabidiol research in the treatment of epilepsy.

Subsequently, other studies by Carlini et al. have reinforced CBD's therapeutic potential, a non-psychoactive phytocannabinoid and, therefore, with fewer side effects than THC, in the treatment of epileptic conditions (33–35). Since then, different researchers have confirmed the pioneering studies of Carlini et al. since the 1970s, that is, that CBD can be a safe and effective therapeutic alternative for the treatment of epilepsy, a condition that affects millions of people across the world (11, 36–40). This contribution made an extensive article published recently (2020) in The NY Times about CBD, which considers Carlini as the “discoverer” of the use of this compound in epilepsy (41). A recent and simple search made based on scientific articles PubMed, using the descriptors “cannabidiol” and “epilepsy” lists ~470 publications addressing this topic. Although there is now much more evidence from studies in animals than in humans, in addition to few randomized controlled trials (28, 42), many clinical observations have suggested cannabinoids, not just CBD, as a new treatment for refractory epilepsies (for better visualization of the contribution of Carlini et al., see Figure 1).

## “To A New Treatment for Refractory Epilepsies:” Clinical Confirmation and The Role of Anecdotal Reports

The fact that medical cannabis is still illegal in several countries, coupled with the high financial cost for patients, when available, ends up favoring the use of artisanal extracts. In turn, these extracts do not always have strict quality control over the quantity and quality of the components present in the formulation. This situation makes it difficult to obtain reliable scientific data regarding the efficiency and safety of the drug use of cannabinoids. However, it is precisely anecdotal reports, mostly obtained through the use of cannabis herbal extract oil, that have provided a considerable amount of evidence for the use of cannabis and CBD in isolation as a treatment for epilepsies (43).

In a study of 74 children resistant to traditional treatment of epilepsy, treatment with CBD-rich herbal cannabis extract (20: 1 THC; ~10 mg/kg CBD per day) reduced the frequency of seizures by 89% of the studied population, with 43% of these children had a reduction that surpassed 50%. Only 6.7% of these children worsened in seizures, with treatment discontinue (44). In another prospective open-label study, using CBD-rich herbal cannabis extract (50: 1 THC; ~13 mg/kg CBD per day) to treat 20 children with Dravet's syndrome for 20 weeks, there was a reduction of more than 70% in seizures (45). Also, about the effectiveness of treatments using artisanal cannabis preparations, a retrospective study investigating the effects of CBD oil in 108 children with epilepsy is highlighted. In this study, 10% of children treated with CBD oil had no seizures, and 39% had a reduction more significant than 50% of those seizures, showing promising potential for this type of preparation. Less than 4% had sedation as an adverse effect (46). These studies, together, point to a direction and show that controlled clinical tests using different cannabinoid compounds, including standardized ones, are necessary, as well as they are promising in the treatment of epilepsy.

In this sense, some clinical studies have been done with standardized cannabinoid compounds, therefore, with better possibilities for the treatment's efficacy and safety. Among these, we highlight the open-label trial by Devinsky et al., which showed the effectiveness of Epidiolex in the control of refractory epilepsy in a study involving 162 patients. These patients received CBD 2 at 50 mg/kg per day, in stages, for 12 weeks. There was a reduction of ~36% in seizures compared to baseline. Mild adverse effects involving drowsiness, reduced appetite, diarrhea, fatigue, and seizures were reported in 79% of patients. Another 12% of patients had serious adverse effects (47). Besides, quality of life was measured in 48 of the 162 initial participants. There was an improvement in the scores obtained through the Quality of Life in Childhood Epilepsy (QOLCE) (48). In short, treatment with CBD proved to be relatively safe, reducing seizures and promoting an improvement in patients' quality of life (47, 48).

Facing the need for a double-blind/randomized/placebo-controlled trials, Devinsky et al. developed a study that reinforced the effectiveness of Epidiolex in the control of epilepsy in patients with Dravet's syndrome. The study included 120 subjects, including children and young adults, who were randomized to receive CBD (Epidiolex) at a dose of 20 mg/kg per day or placebo over 14 weeks. After treatment, 5% of participants who received CBD were free from seizures than the placebo group (0%). Also, 43% of patients treated with CBD had a 50% reduction in seizures, against 27% in the placebo group. It is also important to note that 93% of patients who received CBD treatment had an adverse effect, with the majority (89%) of these effects being considered mild or moderate (e.g., diarrhea, vomiting, drowsiness, etc.) (49). These results reinforce the evidence from the same group in previous studies (47, 48).

In another double-blind, placebo-controlled trial, two groups, one with 86 and 85 patients with Lennox-Gastaut syndrome, were treated, respectively, with CBD or placebo for 14 weeks. In this study, the group that received CBD, 20 mg/kg per day, had an average monthly reduction in epileptic seizures of around 43%, while the placebo group had an average of 21%. Mild and moderate adverse effects occurred in 86% of patients treated with CBD vs. 69% of patients in the placebo group, with diarrhea, drowsiness, decreased appetite, pyrexia, and vomiting being the most frequent. This study points to CBD as useful in treating seizures associated with Lennox-Gastaut syndrome and being relatively well-tolerated, as it has not caused severe adverse effects (50).

A point that has been discussed in the clinic and, therefore, deserves to be highlighted concerns the possible interactions of CBD with other anticonvulsant agents through the so-called polytherapy, so common in patients with refractory epilepsies. A better understanding of this adjunctive therapy is necessary so that there is greater clarification concerning the adverse effects, quality of life of the patient, as well as the effectiveness of the doses used. This understanding should involve, for example, genetic, pharmacodynamic, and pharmacokinetic issues that influence the effects of CBD in the presence of other drugs (and vice versa), thus providing greater security in choosing an appropriate pharmacotherapeutic strategy [for a more detailed review, see (51)]. One of these possibilities that have been documented is the interaction of CBD with benzodiazepine clobazam. Both are metabolized by the cytochrome P450 (CYP) pathway, and CBD could be potentiating the effects of clobazam by inhibiting this metabolism pathway. Likewise, clobazam could also be potentiating the antiepileptic effects of CBD by inhibiting its degradation pathway. This possibility raised a question about the effectiveness of CBD in the treatment of epilepsy, which was: would CBD have effects *per se* in the treatment of epilepsy, or would it need to be associated with clobazam? (52, 53) Although this answer is not yet clear, CBD in Europe has been approved only as an adjunctive treatment with clobazam. However, a study carried out with Lennox-Gastaut syndrome patients and Dravet syndrome who received CBD in the absence of clobazam (54), in addition to other studies (55, 56), strongly point to the fact that this phytocannabinoid exerts its therapeutic effects independently of its interaction with the mentioned benzodiazeinic. Therefore, the evidence suggests that the European Medicines Agency Public Assessment Report's prescription restriction is not supported. The lack of randomization for studies involving CBD interaction with clobazam may have contributed to some misconceptions (52).

In recent years, there have also been some systematic reviews of clinical trials, including meta-analysis, which have reinforced the effectiveness and safety of CBD or CBD-Rich Cannabis Extracts in the treatment of epilepsies (18, 40, 57). After a long time where anecdotal reports predominated, the evidence through well-conducted clinical studies indicates a safe use of cannabinoids, especially CBD, in the treatment of epilepsies. Further studies are needed to understand better the benefits to the possible risks of using cannabinoids in this situation. Studies are needed to better elucidate another prevalent issue among anecdotal reports, which concerns the interaction between CBD and THC influencing antiepileptic effects' effectiveness (20, 58). Would CBD alone be more effective, or would it need to interact with THC and other constituents of *Cannabis sativa*? Such an issue will be further discussed in the next section of this article.

## The Possibility of The Entourage Effect: What Is Known About?

“One plus one is always more than two,” phrase of the song “O sal da terra” by Beto Guedes, a singer of Brazilian popular music. Obviously, he was not referring to cannabinoids but to the need to bring people together to build a better world. The same phrase can also make sense when speculating about the entourage effect observed in many studies that address the therapeutic application of cannabinoids.

The entourage effect is a term suggested referring to a situation where a group of endogenous compounds similar to eCB, when acting together, potentiate the effects mediated by cannabinoid receptors. This term was first mentioned by Bem-Shabat et al. (59), and soon expanded to also define the synergistic effects observed through the use of mixtures of plants in general, including concerning the different compounds present in cannabis. It is worth mentioning in this topic another pioneering aspect of Carlini and his group. Long before the advent of this terminology on cannabinoid effects, the Brazilian group published studies showing the relevance of the interaction between the different phytocannabinoids present in cannabis samples (60–62). Thus, evidence indicates that many of the therapeutic effects observed through the use of phytocannabinoids occur, in fact, much more from the complex and poorly understood interaction of all the compounds present in the plant (mainly THC and CBD) rather than the isolated action of a single compound (63, 64). In this case, can one plus one also be more than two?

Studies, mainly in animals but also in humans, have shown that the answer is yes. A recent meta-analysis study published by Pamplona et al. (40), which searched 199 articles (with 11 validated references) with a total of 670 patients, showed that CBD-rich extracts seem to present a better therapeutic profile than purified CBD. In this meta-analysis, 71% of patients treated with CBD-rich extracts reported some improvement in their epileptic condition, compared to 46% of patients treated with purified CBD. Patients treated with CBD-rich extracts also required lower doses (6 mg/kg/day) than patients who used only CBD (25.3 mg/kg/day), that is, an effect four times greater on the part of the unpurified extract. Still, in the same study, it was also possible to notice that patients treated with CBD-rich extracts had fewer side effects, both mild (33%) and severe (7%) when compared to those who received purified CBD (76% mild and 26% serious).

In addition to the main constituents of cannabis CBD and THC, another possibility that favors the entourage effect is the findings of the anticonvulsant potential of other phytocannabinoids, for example, delta-9-tetrahydrocannabivarin (THCV) and cannabidivarin (CBDV). These phytocannabinoids also proved useful anticonvulsants, with THCV having its effects *via* CB1 receptors, while CBDV did not (65–67). CBDV also had their anticonvulsant effects enhanced by CBD (66). Additional contributions to these specific topics through Carlini-trained researchers can be found in recent reviews (68–71). Considering the spectrum of possibilities of cannabis expanded to more than 200 terpenes present in the plant, the findings can be even more promising. Some terpenes are known to have pharmacological activities in the central nervous system, although they have not been tested in patients with epilepsy (63, 72). Although such interactions do seem to occur, more controlled clinical studies proving such a possibility are needed. However, regardless of whether they are better together or apart, one thing is sure, phytocannabinoids are proving to be increasingly influential in the treatment of epilepsies.

## If Cannabidiol Works, How Does it Work?

To speculate about the neurobiological mechanisms involved in the antiepileptic activity of cannabinoids, including cannabidiol, it is necessary to understand a little more about the eCB system's physiology. This fact has been well-elucidated since the early 90s, when the discovery of this system occurred, which revolutionized the understanding of many neurophysiological responses. The eCB system consists of two receptors (CB1 and CB2), endogenous ligands (anandamide/AEA and 2-arachidonylglycerol/2-AG), and enzymes involved the synthesis and degradation of these ligands. It is a system with neuromodulatory functions, which regulate the presynaptic release of both excitatory and inhibitory neurotransmitters (73, 74). These neuromodulatory functions appear to play an essential role in controlling epilepsies, for example, through the activation of CB1 type cannabinoid receptors (75).

These receptors are located presynaptically, and their activation, either by endogenous ligands (e.g., AEA) or exogenous (e.g., THC), results in a transient hyperpolarization of the presynaptic membrane that, consequently, inhibits the release of excitatory neurotransmitters like glutamate (76). This fact agrees with the evidence that shows the downregulation of CB1 receptors in axial glutamatergic terminals extracted from the brain tissue of patients with epilepsy. On the other hand, the evidence also points to the upregulation of the same receptors at the GABAergic axonal terminals. In both possibilities, there is a loss of control over neuronal hyperexcitability, favoring epilepsy. The antiepileptic effects obtained from manipulating the eCB system or using exogenous phytocannabinoids may be related to the reestablishment of control over this hyperexcitability (19, 77–79).

In the case of phytocannabinoids, THC has a high affinity for the CB1 receptor and, through this receptor, can regulate neuronal excitability. This compound was the first phytocannabinoid to have its anticonvulsant properties evaluated, which must result from a reduction in the levels of excitatory neurotransmitters caused by its agonist action on CB1 receptors (19, 20). While CBD, the plant's non-psychoactive phytocannabinoid and possibly the most studied when it comes to antiepileptic properties, has a mechanism of action that has not yet been elucidated. This phytocannabinoid has a low affinity for CB1 receptors and may have its antiepileptic effect related to neuronal excitability's modulation through changes in the influx of Ca and Na ions, as well as actions in vanilloid receptors, adenosinergic and serotonergic systems (80–82). Another possibility to explain CBD's antiepileptic effects would be an eventual ability to inhibit both uptake/hydrolysis of the eCB and, thus, indirectly, to decrease neuronal excitability (83) by potentiating this system (84, 85). The fact is that this issue of the neurobiological mechanisms involved in the antiepileptic action of cannabinoids is not entirely defined and still requires a better understanding [for a complete review of the possible mechanisms of action of cannabinoids in epilepsy, see (80)]. As science grasps these mechanisms, this will result in more efficient pharmacotherapeutic approaches for the treatment of epilepsy and make it possible to expand the medicinal use of cannabinoids, including CBD, for other neuropsychiatric diseases.

## Can The Success of Cannabidiol In The Treatment of Epilepsy Predict The Same Path for The Treatment of Neuropsychiatric Disorders?

It is not yet possible to say whether the use of CBD and other cannabinoids to treat different neuropsychiatric disorders will follow the same route observed for the treatment of epilepsy. However, significant steps have also been taken for these other possibilities. From depression to anxiety, including PTSD, addiction, neurodegenerative diseases, and ASD, these are disorders that, according to some studies, can use cannabinoids, especially CBD, as a pharmacotherapeutic alternative (4, 8, 86–88).

### Depression

Regarding depression and anxiety, it is known that many cannabis users report its use for its relaxing effects; therefore, as a way to reduce the symptoms of these disorders (87, 89, 90). Additionally, several studies have pointed to the potentization of the eCB system or the use of exogenous ligands as promising possibilities in treating depression (5, 7, 91–97). Reinforcing this possibility, the blockade of this system, whether through the use of antagonists or genetic deletion, seems to lead to depressive and anxiety symptoms, which caused the withdrawal of the CB1 antagonist rimonabant, proposed for the treatment of obesity, from the market (98–100). According to this perspective, patients with major depression had reduced serum levels of eCBs, in addition to a lower density of CB1 receptors in the glial cells of the brain gray matter (101, 102). In this sense, a proposal to reestablish the eCB system's functions by inhibiting the degradation of its endogenous ligands can be explored as an antidepressant potential (103). Remembering that CBD, a phytocannabinoid with few side effects, may be acting in this way, potentiating the eCB system (84), even being reported in different studies as an effective antidepressant (6, 104, 105). Considering that traditional antidepressants (serotonin and/or noradrenaline reuptake inhibitors) have relatively low efficiency and still need weeks for their effects (106), it is suggested that the manipulation of the eCB system, which even has a response rate faster, can be an alternative for the treatment of depressive disorders [for a more detailed review, see (7, 91, 93, 96)].

### Anxiety

About anxiety, CBD has also been shown to be a more exciting alternative, given the potentially anxiogenic effects of THC (107, 108). Several pre-clinical studies using different animal models (109–116), as well as some clinical studies (117–121), confirm the anxiolytic effects of CBD (122). In this research area, it is worth mentioning the vital participation of groups from the Faculty of Medicine of Rib. Preto—University São Paulo, BR, led by Zuardi and Guimaraes. In addition to the use of CBD, manipulation of the eCB system is an alternative in treating anxiety. This system is located in brain regions important for modulating responses related to fear and anxiety (123), with increased anandamide *via* inhibition of its degradation, promoting anxiolytic effects (109, 124–127). Considering the high abuse potential of benzodiazepines and the slow response of selective serotonin reuptake inhibitors (SSRIs), both CBD and potentiation of the eCB system are promising alternatives in pharmacotherapy of anxiety disorders [for a mor detailed review, see (87, 122, 127, 128)].

### PTSD

Until recently considered as an anxiety disorder, post-traumatic stress disorder (PTSD), which from the DSM-5 was included in a new category called “trauma and stress-related disorders,” has also responded very well to research that involves cannabinoid treatment, especially CBD (8). The speculations started from the work of Marsicano et al. (129), showing the eCB system's role in the extinction of aversive memories. From then on, a series of pre-clinical studies started to indicate that the potentiation of the eCB system (130), the use of exogenous agonists for the CB1 receptor (131) or even the CBD (109, 132) could facilitate the extinction of aversive memories. In addition to facilitating the extinction process, different studies have shown the effect of cannabinoids impairing the processes of retrieval and consolidation of these memories, that is, more possibilities for intervention in the remembrance of traumatic events (133–135). In the face of so many reports of pre-clinical studies, it was not long before evidence also emerged from clinical studies (119, 136–140) and thus reinforced the potential of cannabinoids, including CBD, as a therapeutic alternative for the treatment of this disorder [for a more detailed review, see (8, 141–145)].

### Addiction

In addition to PTSD, another neuropsychiatric condition where memories play a fundamental role, and that there is also evidence for the use of cannabinoids, is addiction/relapse to drugs of abuse. Although it seems to be a paradoxical variant, understanding the action of cannabinoids in the breakdown of hedonic or reinforcing memories can provide up-and-coming therapeutic alternatives. In this perspective, de Carvalho and Takahashi showed in a pioneering way the inhibitory effect of CBD in reactivation sessions in animals that previously had conditioned place preference induced by morphine or cocaine (88). This finding suggests CBD's disruptive effect on the reconsolidation of memories associated with drugs of abuse, thus reducing the risk of relapse (146). A similar result was reported by Luján et al. (147), showing that the CBD attenuated cocaine-induced conditioned place preference, in addition to reducing voluntary consumption by mice. Besides, this work showed that CBD increased the expression of CB1 receptors and neural cell proliferation in the hippocampus, reinforcing the ability of this cannabinoid to modulate both behavioral and molecular manifestations related to cocaine reinforcement (147). Cannabinoid receptors CB1 and CB2 even seem to perform opposite functions, and the antagonism of CB1 receptors has the same inhibitory effects seen in the activation of CB2 receptors concerning the modulation of cocaine-induced sensitization and conditioned place preference (CPP). These effects probably occur due to a block in neuronal activation of the hippocampus (148). Other studies have also shown the CBD's ability to also reduce alcohol consumption in animal models of an alcohol use disorder, in addition to reducing alcohol-related steatosis and fibrosis in the liver, and alcohol-related brain damage, preventing neuronal loss (149). From a clinical perspective, promising results showed that the voluntary use of cannabis caused a decrease in crack use and also promoted an improvement in the quality of life in individuals dependent on this substance (150–152). The evidence from CBD as a treatment for drug abuse disorders is still discreet but deserves a closer look [for a more detailed review, see (10, 149, 153)].

### NDDs

One possibility that is increasingly attracting researchers' attention to the use of cannabinoids is related to the application of these compounds in neurodegenerative disorders (NDDs). These NDDs are strongly related to oxidative damage and a series of neuroinflammatory responses that ultimately lead to cell death (154). Among the NDDs, the most common are Parkinson's disease (PD) and Alzheimer's disease (AD), conditions in which the potentiation of the eCB system (155) or even the use of phytocannabinoids, especially CBD (156, 157), can play an auspicious role as neuroprotectants (4). This promising possibility on CBD is pointed out *in vitro* and *in vivo* studies (158–160), and even in clinical studies (161). Taking into account that the current classic treatments for NDDs do not stop and/or slow the progression of the disease, alternatives such as CBD or any other substances that target the eCB system can be good candidates as prototypes for the development of neuroprotective drugs [for a more detailed review, see (154–157, 161)].

### ASD

Another neuropathology that appears to be associated with inflammatory processes and, therefore, can also be a target for cannabinoids is an autism spectrum disorder (ASD) (86, 162). This disorder is characterized by constant communication and social interaction deficits and restricted and repetitive behavior patterns, which still have unknown etiopathogenesis (163). One of the possibilities may be an imbalance in the eCB system, responsible for regulating some typically impaired functions in the ASD (164–168). This fact, associated with the anti-inflammatory activity of cannabinoids, has encouraged pre-clinical (169–171) and clinical (12, 172, 173) research to investigate the therapeutic potential of cannabinoids for the treatment of ASD. Among the possibilities, CBD seems to be the safest and most promising alternative (12, 172, 173), although other phytocannabinoids like CBDV also present themselves as candidates (171). The reestablishment of the balance of the eCB system and the anti-neuroinflammatory activity seems to support these compounds' activities as a treatment to ASD [for a more detailed review, see (86, 163, 164, 166, 174)].

There are still many other possibilities for neuropsychiatric disorders that can find cannabinoids as a possible therapeutic option. In this case, CBD, the main focus of this review, and other phytocannabinoids (e.g., THC, THCV, CBDV) appear to present quite promising pharmacotherapeutic alternatives for an increasingly broad number of neuropsychiatric disorders. However, for all these possibilities, including those mentioned here, further prospective, double-blind, placebo-controlled studies must be needed (for a summary, see Table 1). These studies are essential to ensure the effectiveness and safety of these compounds in each specific situation. In any case, this is a promising field of study where many pharmacotherapeutic alternatives may be revealed.

**Table 1:** CBD as a promising psychopharmacological alternative in the treatment of neuropsychiatric disorders.

**Neuropsychiatry Disorders**	Briefly, What Is Known About The Use of Cannabidiol in This Condition^*^	**For a More Detailed Review, See:**
Depression	Several studies, preclinical and clinical, suggest that the eCB system's blockade induces depressive-like responses, while the potentiation of this system produces an antidepressant action. Therefore, it is suggested that substances that directly activate cannabinoid receptors or promote the increase of their endogenous ligands, such as CBD, may represent good therapeutic alternatives for the treatment of depression.	(7, 91, 93, 96)
Anxiety	For the treatment of anxiety, CBD is the most promising alternative from the plant, given THC's anxiogenic effects. Another alternative is the potentiation of the eCB system by inhibiting the degradation and reuptake of anandamide. Both preclinical and clinical studies have pointed to the possibility that cannabinoids can be used as anxiolytics.	(87, 122, 127, 128)
Post-traumatic stress disorder (PTSD)	The eCB system plays a fundamental role in the extinction of aversive memories; therefore, its enhancement facilitates this process. Additionally, this system's enhancement can still block the retrieval and reconsolidation processes of this type of memory. In all possibilities, the result seems to be the improvement of symptoms related to PTSD, both in preclinical and clinical studies. In this sense, CBD stands out as an up-and-coming alternative.	(8, 141–145)
Addiction	Although it seems paradoxical, cannabinoids act by causing a breakdown in hedonic or reinforcing memories related to drugs and can be an alternative in treating addiction/relapse. Pre-clinical studies have shown CBD's effects in reducing drug-seeking behavior in models involving morphine, cocaine, and alcohol. Studies show that cannabis use can lead to reduced consumption and improved quality of life in crack-dependent individuals from a clinical perspective.	(10, 149, 153)
Neurodegenerative diseases (NDDs)	Both the enhancement of the eCB system and phytocannabinoids have effects in reducing oxidative stress and neuroinflammation, conditions present in NDDs. *In vitro, in vivo*, and clinical studies suggest a neuroprotective action of CBD, making this phytocannabinoids a therapeutic possibility for treating diseases such as Parkinson's and Alzheimer's.	(154–157, 161)
Autism spectrum disorder (ASD)	Although the etiopathogenesis of ASD is unknown, evidence points to an imbalance of the eCB system and the presence of a neuroinflammatory process. From this perspective, phytocannabinoids, especially CBD, appear with good preclinical and clinical evidence to improve symptoms in ASD, possibly through reestablishment of the eCB system and already confirmed anti-neuroinflammatory activities.	(86, 163, 164, 166, 174)

* *Important Clinical studies regarding Cannabidiol as a treatment for these neuropsychiatric disorders are still very preliminary or even non-existent. However, the evidence found is promising and suggests that investing in larger-scale placebo-controlled clinical studies is necessary and worthwhile*.

## Conclusion and Future Perspectives

In conclusion, we emphasize promising pre-clinical and clinical findings with cannabinoids, mostly from Brazilian-based researchers and other researchers worldwide. Specific studies have focused on the multifunctional phytocannabinoid, CBD, showing remarkable benefits, mainly for refractory epilepsy in children. These data contributed to the considered “prohibited substance” to enter the list of medicines for controlled use by the National Health Surveillance Agency (ANVISA), the regulatory agency responsible for the approval of new drugs in Brazil. Besides, one may anticipate other phytocannabinoid-based preparations and even new drugs acting at the endocannabinoid system as a promising therapeutic advance for other neuropsychiatric disorders, represented here by depression, anxiety-related disorders, PTSD, drug addiction and drug-induced relapse, neurodegenerative disorders, and ASD. If CBD (or other cannabinoids) with regard to neuropsychiatric disorders, will follow the same path observed for refractory epilepsies-from alternative medicine to a new treatment-, only advances in research can respond. While the definitive answers do not arrive, the fact is what we have so far allows us to glimpse a promising path.

## Author Contributions

RB and RT: final form of the manuscript. All authors: conceived the review and prepared the first draft.

## Conflict of Interest

The authors declare that the research was conducted in the absence of any commercial or financial relationships that could be construed as a potential conflict of interest.

## Abbreviations

eCB: endocannabinoid
PTSD: post-traumatic stress disorder
ASD: autism spectrum disorder
THC: delta-9-tetrahydrocannabinol
CBD: cannabidiol
QOLCE: quality of life in childhood epilepsy
THCV: delta-9-tetrahydrocannabivarin
CBDV: cannabidivarin
CB1: cannabinoid receptor type 1
CB2: cannabinoid receptor type 2
AEA: anandamide
2-AG: 2-arachidonylglycerol
GABA: gamma-aminobutyric Acid
SSRIs: selective serotonin reuptake inhibitors
DSM-5: Diagnostic and Statistical Manual
CPP: conditioned place preference
NDDs: neurodegenerative disorders
PD: Parkinson disease
AD: Alzheimer disease
ANVISA: National Health Surveillance Agency.
